# Even affective changes induced by the global health crisis are insufficient to perturb the hyper-stability of visual long-term memory

**DOI:** 10.1186/s41235-022-00417-2

**Published:** 2022-07-16

**Authors:** Chong Zhao, Keisuke Fukuda, Sohee Park, Geoffrey F. Woodman

**Affiliations:** 1 Department of Psychology, University of Chicago, Chicago, IL 60637 USA; 2 Institute for Mind and Biology, University of Chicago, Chicago, IL 60637 USA; 3 Department of Psychology, University of Toronto Mississauga, Mississauga, ON L5L 1C6 Canada; 4 Department of Psychology, University of Toronto, Toronto, ON M5S 3G3 Canada; 5 Department of Psychology, Vanderbilt University, PMB 407817, 2301 Vanderbilt Place, Nashville, TN 37240-7817 USA

## Abstract

Past studies of emotion and mood on memory have mostly focused on the learning of emotional material in the laboratory or on the consequences of a punctate catastrophic event. However, the influence of a long-lasting global condition on memory and learning has not been studied. The COVID-19 pandemic unfortunately offered a unique situation to observe the effects of prolonged, negative events on human memory for visual information. One thousand online subjects were asked to remember the details of real-world photographs of objects to enable fine-grained visual discriminations from novel within-category foils. Visual memory performance was invariant across time, regardless of the infection rate in the local or national population, or the subjects’ self-reported affective state using the Positive and Negative Affect Schedule (PANAS). Thus, visual memory provides the human brain with storage that is particularly resilient to changes in emotional state, even when those changes are experienced for months longer than any imaginable laboratory procedure.

**Supplementary Information:**

The online version contains supplementary material available at 10.1186/s41235-022-00417-2.

## Significance statement

The global health crisis has impacted the brain by increasing the experience of negative human emotions and making memory storage more difficult. However, cognitive psychology suggests that visual representations stored in long-term memory may be particularly useful during such times in human history because our memories for visual details might not be impaired by chronic stress in the same way that memory for other types of information has been shown to be. Consistent with this hypothesis, we found that human subjects’ memory for particular exemplars of objects was unaffected by the severity of the pandemic at the time of data collection, and unrelated to the perturbation of the subjects’ self-reported emotional state.

## Introduction

Negative emotions are believed to influence the operation of our memory systems. Laboratory-based studies suggest that the temporary induction of negative emotions results in the recall of both less and different information than when a positive emotional state is induced (Levine & Burgess, 1997). In contrast, singular catastrophic events such as the John F. Kennedy assassination, or the 9/11 terrorist attack, have provided a snap shot of the impact of population-wide negative emotions on memory. Such flashbulb-memory studies focus exclusively on the autobiographical memory surrounding the short lived, but widely experienced event (Hirst et al., 2015). Thus, we do not yet understand of the role of chronic, society-wide negative conditions on memory and learning.

The global pandemic has raised important questions in cognitive science due to its triggering unprecedented changes in the way we live, learn, and teach. Parents are concerned about the impact of pandemic-induced negative emotions on learning. College students, forced to move to online learning environments, worry about retention of knowledge. Moreover, there are increasing reports of mental illness across the world especially with respect to depression, anxiety, and stress (Dean et al., 2021). It is therefore of utmost importance to document long-term memory during the pandemic. Has the pandemic-induced negative mood changed our ability to memorize and retain complex information over time?

Research suggests that visual long-term memories may be particularly robust (Brady et al., 2013), and that the detail is visual storage may increase during negative emotional states, unlike semantic memories or memories derived from other sensory modalities (Kensinger et al., 2007). Here, we ask whether visual long-term memory was subject to changes in the quality of the representations it stored in reaction to the large shifts in emotional state that accompanied the different phases of the COVID-19 pandemic that unfolded during the year 2021.

**Fig. 1:**
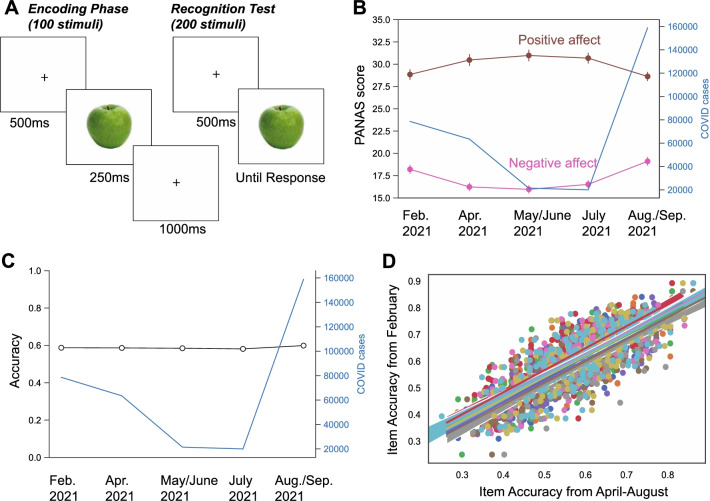
Visual recognition memory was highly stable despite large changes in pandemic state and perceived emotional states. **A** Adult participants (200 in each data collection, 1000 total) were asked to remember 100 pictures, with 5 exemplars from 20 semantic categories. Each picture was shown for 250 ms during the encoding phase. They were later tested by recognizing the studied pictures from a stream of 200 pictures, consisting of all 100 studied pictures and 100 new pictures with the same number of exemplars from the same semantic categories. **B** COVID-19 case numbers influence on subject’s self-reported positive and negative affect. **C** Recognition memory performance (proportion correct) did not change with respect to the time of data collection, even as COVID-19 cases in the US varied drastically. The mean, variance, and skewness of the d-prime index of memory sensitivity were also statistically unchanged across the five data collections (see Additional file 1: Fig. S1). **D** Pairwise correlations between February recognition accuracy of each item and recognition from each other data collection period

Cases of infection during the COVID-19 pandemic, and the associated secondary impacts of the pandemic, have induced negative emotions into the lives of people all over the world during the past two years. Our goal in this study was to leverage this unfortunate situation to determine if visual long-term memory storage is influenced by the large-scale change in subjects’ emotional state induced by the global pandemic at the time of data collection, as well as whether visual long-term memory storage exhibits any shift in which objects are remembered during the different phases of the pandemic during 2021. Five 200-person groups of subjects were sampled across five time-windows extending from February of 2021 to September of 2021. We sampled individuals living in the United States of America where pandemic stressors included both heavy circulation of the virus and loss of life, as well as social and economic upheaval. Each subject studied 100 real-world objects drawn from 20 distinct semantic categories (Brady et al., 2008). Note that we designed this task to be sufficiently difficult so that subjects’ performance would not be at ceiling and our task would require the kind of demanding highly detailed visual discriminations that result in errors in our daily lives. After a 5-min rest period, we tested subjects’ memory by showing them 200 test objects with half being studied and the other half novel (Fukuda & Woodman, 2015) (Fig. 1A). Subjects also completed a 20-question Positive and Negative Affect Schedule (PANAS) questionnaire so that we could verify that our subjects were in fact experiencing emotional impacts of the pandemic (Watson, 1988).

It is possible to predict both better and worse memory performance as a result of the pandemic. Chronic stress is believed to have a negative influence on a wide range of memory systems. It can trigger an increase in glucocorticoids (de Quervain et al., 2016), and thus impair hippocampus-dependent memory and learning (Bangasser & Shors, 2007), as well as interfering with amygdala-regulated emotional memory formation (Roozendaal et al., 2009). Stress-induced neural impairments have been shown to result in lower working and long-term semantic memory in human adults (Shields et al., 2017). Human participants also perform better on working memory tasks when they rate their affect as more positive (Brose et al., 2014). These pieces of evidence suggest that we will see memory is worsened during the pandemic. In contrast, a number of other studies in the literature suggest that negative emotions may have faciliatory effects on memory. Specifically, previous reports suggest that negative emotions can increase the precision of our visual memory representations (Xie & Zhang, 2016, 2017), although these findings have not been without controversy (Brose et al., 2014; Souza et al., 2021). Thus, the literature provides motivation for both predicted impairment and facilitation as a function of the intensity of emotions induced by the pandemic crisis.

## Methods

We first estimated the power necessary to detect differences between groups using long-term memory recognition tests. Using an effect size of 0.4 derived from previous studies of visual memory (Milliken & Jolicoeur, 1992), and significance level of 0.05, we estimated that we needed 200 subjects to achieve power of 0.8 in our experimental design (Faul et al., 2007). We collected data from 1000 subjects living in the United States of America, 18–35 years of age, across 5 sample periods using the Prolific online system. Subjects were compensated at a rate of $6 for an hour of their time.

After informed consent was obtained for procedures approved by the Vanderbilt Institutional Review Board, every participant first completed a 20-question PANAS questionnaire consisting of 10 questions about positive affect and 10 questions about negative affect (Watson, 1988). We compared our subjects’ responses to established normative data collected during a pre-pandemic sample of 660 healthy college-aged adults (Watson et al., 1988). Then, each participant completed a picture study phase followed by a recognition-memory test phase, using our standard visual long-term memory testing procedures (Fukuda & Woodman, 2015; Zhao & Woodman, 2021). We note that each picture is shown for only 250 ms in this paradigm so that performance is not at ceiling in our task, allowing us to see improvement, if it exists.

During the study phase we presented 100 photographs of real-world objects, with 5 exemplars in each of 20 distinct semantic categories. Mean picture size was approximately 4.6° by 4.6° of visual angle assuming the subject was seated 80 cm from the screen. Each image was centered on the screen during both study and test phases.

During the study phase, each trial started with a 1000-ms fixation cross. Next, a picture was shown at the center of the screen for 250 ms. During the test phase, we showed each subject 200 pictures, one at a time, with half of the pictures being shown in the study phase and the other half being novel to the participant. The 100 novel pictures had 5 exemplars in 20 distinct categories as well. Each trial of the test phase started with a 1000-ms fixation cross, followed by the presentation of the test picture at the center of the screen until the participant made a button press to record their old versus new response, as well as confidence level. There was then a 1000-ms inter-trial interval that followed.

The participants used the numbers on a keyboard to indicate their confidence and whether they thought the test stimulus was old or new. The number keys 1 and 2 indicated that the item was old, with high and low confidence levels, respectively. The number keys 9 and 8 indicated that the item was new, with high and low confidence level, respectively. The stimuli were drawn from a published set of real-world objects (Brady et al., 2008). Both parts of the experiment were programmed using jsPsych package (De Leeuw, 2015). To promote open science practices, materials and data are available through Open Science Framework (https://osf.io/hywkt/). Participants were excluded from the averages if they had failed more than 20% of the attentional checks during the online experiment, or if they failed to respond on more than 10% of the memory tests. This resulted in a rejection of 5–8% of participants across our data collection epochs.

The COVID case numbers were calculated as the mean of the 7-day rolling average of Johns Hopkins Coronavirus Resource Center data centered on each data collection period. We calculated the mean and variance of the memory performance using the np.mean() and np.var() function, respectively, in python. To determine the relative evidence for an against the null hypothesis, we calculated JZS Bayes’ factors (Rouder et al., 2009), in which the BF_10_ indicates how much more likely the alternative is over the null, and BF_01_ how much more likely the null is over the alternative.

In externally validating the stability of memorability across our data collection, we used a pre-trained multi-layer convolutional neural network to acquire the memorability score of each individual stimulus. That is, the deep neural network, Resmem, is a pre-trained convolutional neural network that was trained to predict memorability of individual images (Needell & Bainbridge, 2021). We applied Resmem directly to our image set used in our experiment, such that the model returned a memorability score ranging from 0 to 1 for each image. The scores were rescaled to 0–100, with higher scores indicating a more memorable image, as predicted by Resmem. The memorability scores, ranging from 0–100, were then correlated to the sensitivity index from our human dataset. The Spearman correlation coefficients provide a measure of the similarity between the machine and human performance-derived memorability from each of our data collection periods.

## Results

First, we empirically validated our assumption that the pandemic had changed our subjects’ emotional state relative to previously established norms. The results from our questionnaire show that subjects’ reported level of negative affect did fluctuate with the case-load level experienced in the United States across the periods of data collection. That is, subjects’ reported level of negative affect differed significantly across time (*F*(4196) = 8.55, *η*^2^ = 0.033 *p* < 0.001, BF_10_ = 259.09, Fig. 1B), with subjects’ self reported positive affect mirroring the negative affect ratings (*F*(4196) = 3.29, *p* = 0.01, *η*^2^ = 0.013, BF_10_ = 58.89, Fig. 1B). Additionally, we compared our measures of positive and negative affect with norms acquired during a non-pandemic baseline period (i.e., the momentary population means of 29.7 ± 7.9 for positive affect and 14.8 ± 5.4 for negative affect). The negative affect scores from all five data collections samples were significantly different than the momentary population mean in the PANAS scale (February 2021: *t*(199) = 6.57, *p* = 0.0001, BF_10_ = 1.86 × 10^7^; April 2021: *t*(199) = 2.89, *p* = 0.0042, BF_10_ = 4.48; June 2021: *t*(199) = 2.32, *p* = 0.021, BF_10_ = 1.08; July 2021: *t*(199) = 3.60, *p* < 0.0004, BF_10_ = 38.47; September 2021: *t*(199) = 8.71, *p* = 0.0001, BF_10_ = 4.80 × 10^12^). In contrast to this pattern, the positive affect scores from the first four data collection periods were not different from the normal momentary value (all *p*s > 0.05, BF_01_ = 1.96–6.54), with the exception of the fifth data collection where the positive affect was below norm (*t*(199) = 2.10, *p* = 0.04, BF_10_ = 0.68). Thus, our subjects’ responses showed that they were experiencing negative emotional effects of the pandemic. Did these changes in affect impact visual recognition memory performance?

Recognition memory performance was stable across our five data collection periods (*F*(4,196) = 1.71, *p* = 0.15, *η*^2^ = 0.007, BF_01_ = 1285.85, Fig. 1C). This stability was observed despite case numbers dropping by 4 times from February 2021 to July 2021, and then increasing again by 8 times from July 2021 to September 2021. We note that this lack of change in memory performance was not due to a ceiling or a floor effect; our subjects’ mean performance was approximately 59%, and significantly above chance (*t*(999) = 38.47, *p* = 0.00001, *η*^2^ = 0.425, BF_10_ = 2.02 × 10^195^). We note that mean performance in this sample is numerically similar to previous studies using this same task while recording brain activity during pre-pandemic research (Fukuda & Woodman, 2015; Zhao & Woodman, 2021).

One possibility that we considered was that subjects’ negative emotions would have a cumulative effect on visual memory such that we would observe a slow deterioration of performance across time during the pandemic. However, there was no reduction in accuracy across our sampling during the year. Next, we analyzed other performance metrics to determine if changes in emotional state had modified psychometric properties other than the mean hit rate of visual recognition memory. However, we found no evidence for an effect of the pandemic on the d’ metric from signal-detection theory (Additional file 1: Fig. S1A), the variance (Additional file 1: Fig. S1B), or the skewness of the distribution of subjects’ memory responses. Collectively, these results showed that visual recognition memory was stable regardless of the emotional state induced by the pandemic.

To determine if a finer grained analysis might find a relationship between emotional state and recognition memory, we collected the zip code of the county of current residency from the subjects in the August 2021 sample. Out of the 200 participants in this sample, 196 subjects responded to this demographic question, and we then searched for the cumulative vaccination rate for each county on Aug. 29th, when the survey was made public online. We found that the vaccine rate (one shot or more) in the county of residence was not significantly correlated with recognition memory accuracy (*r*(195) = 0.10, *p* = 0.18), negative affect (*r*(195) =  − 2.62 × 10(− 5), *p* = 0.99), or positive affect (*r*(195) =  − 0.03, *p* = 0.67). Similarly, the rate of fully vaccination on a county level did not predict recognition memory accuracy (*r*(195) = 0.08, *p* = 0.29), negative affect (*r*(195) = 0.02, *p* = 0.81), or positive affect (*r*(195) =  − 0.03, *p* = 0.70). In sum, we did not find that county vaccination rates significantly modulated recognition memory, showing that we had not simply averaged effects of opposite directions given the political polarization in the United States currently.

Although we found that changes in emotion induced by the pandemic did not affect the mean of recognition memory across all pictures of objects, perhaps which objects people remembered changed across time, with subjects’ remembering some objects better under strong negative emotions, whereas other objects are remembered better when subjects approach them under positive emotional states. Consistent with this alternative explanation for our results, previous research has suggested that memory for individual items may be different and depend on the contextual valence of the item (Hidalgo et al., 2015). If this explanation is correct, then we should see that the memorability of an individual picture changes across time. For instance, a picture of people smiling and talking might be the least memorable item in February 2021, but be highly memorable among subjects after July 4th, when emotional states approached normal levels. Contrary to this prediction, we found that the hit rate of individual items did not change across the five data collection periods. We measured memorability across time by correlating the recognition performance of each individual item in one round of data collection with that in all other rounds of data collections, and we found high correlations of individual item hit rates across all possible pairwise comparisons (Fig. 1D, r(199)s > 0.77, *ps* < 0.00001). More importantly, we did not find higher between-group memorability correlations when the data were collected during periods in which the emotional states were more similar, as would be expected if affect where changing which items were memorable. Next, we applied a neural network trained for predicting item-level memorability to our data to determine if the pandemic had warped which items were memorable (Needell & Bainbridge, 2021). The Spearman correlation between real-world recognition performance of each item, measured by hit rate minus the false alarm rate, and neural net predicted memorability score, was positive and highly similar across all of our data collection periods (Feb. 2021: *r*(199) = 0.24, *p* < 0.0006; Apr. 2021: *r*(199) = 0.17, *p* = 0.02; Jun. 2021: *r*(199) = 0.22, *p* = 0.002; Jul, 2021: *r*(199) = 0.16, *p* = 0.02; Aug. 2021: *r*(199) = 0.25, *p* < 0.0003). Thus, our findings show that pandemic-induced emotional states did not affect the item-level memorability, as validated by both human recognition memory data and neural network modeling of item-level memorability.

**Fig. 2:**
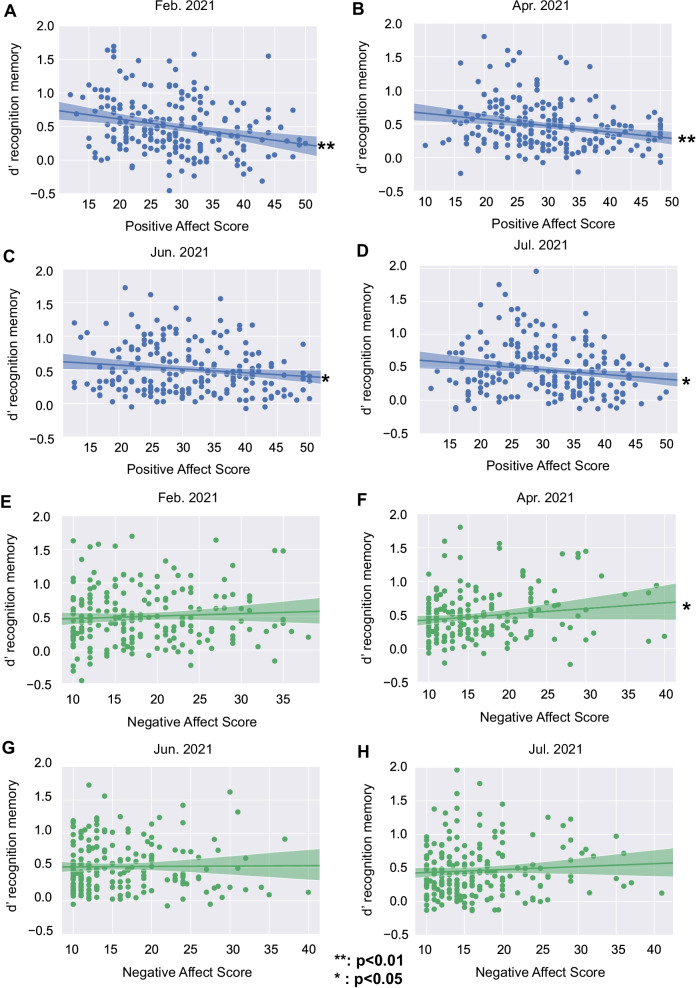
Positive effect scores across all four data collections, but not negative affect scores, were negatively correlated to recognition memory performance. **A**–**D** Positive Affect score was significantly negatively correlated with d prime index in Feb. 2021 (*r*(199) =  − 0.26, *p* = 2.7 × 10(− 4)), Apr. 2021 (*r*(199) =  − 0.22, *p* = 1.7 × 10(− 3)), Jun. 2021 (*r*(199) =  − 0.14, *p* = 0.04) and Jul. 2021. (*r*(199) =  − 0.16, *p* = 0.02). **E**–**H** Negative Affect socre, on the contrary, was generally not significantly correlated with the d prime index in Feb. 2021 (*r*(199) = 0.062, *p* = 0.38), Jun. 2021 (*r*(199) = 0.011, *p* = 0.88) and Jul. 2021. (*r*(199) = 0.073, *p* = 0.31). The only exception was in Apr. 2021, when negative affect was positively correlated with d prime (*r*(199) = 0.15, *p* = 0.04)

We did observe a relationship between emotional state and visual memory that was stable across all periods of data collection. Within each data collection, we found that positive affect was negatively correlated to the recognition memory performance (Fig. 2A–D), while the negative affect was not predictive of recognition memory performance (Fig. 2E–H). This may seem counterintuitive at first, though we found that people with high positive affect also tended to have more false alarms during the task (Feb. 2021: *r*(199) = 0.19, *p* = 0.0076; Apr. 2021: *r*(199) = 0.35, *p* < 0.00001; Jun. 2021: *r*(199) = 0.35, *p* < 0.00001; Jul, 2021: *r*(199) = 0.27, *p* < 0.0001; Aug. 2021: *r*(199) = 0.14, *p* = 0.04). Because positive affect remained normal despite variability in the pandemic-induced negative affect level, we believe that high positive affect may induce lower decision boundaries in recognition memory (Ratcliff et al., 2016), so that people were more likely to falsely report a memory of a non-studied item. Moreover, the null correlation between negative affect and recognition memory across individuals within the same data collection period provided additional support to our claim that visual recognition memory was not influenced by affect changes in general.

## Discussion

In summary, we found that people remembered pictures of common objects just as well regardless of their emotional state during the height of the pandemic in 2021. Our data also indicate that the same objects were most memorable whether the local or national environment was awash in COVID cases or was in a relatively safe period.

We found that the pandemic-induced changes in the emotional states of our participants had no effect on their ability to store visual memories during the last year. These findings are consistent with previous work suggesting that changes in emotional state induced in the laboratory are unrelated to how well subjects remember visual memoranda (Souza et al., 2021). There is another possibility. It is possible that the apparent stability of memory performance is a combination of effects that negate each other. In the Introduction, we presented the competing ideas that chronic stress impairs memory while negative emotion improves memory. It could be that the stable function we observed was due to a tradeoff of these two effects. This would seem unlikely as these two effects would need to have identical, mirror-reversed time courses over the chaotic events of 2021. Because we cannot empirically address this possibility here, we believe this is an intriguing question for future study.

## Supplementary Information

**Additional file 1**.** Fig. S1**. Visual recognition memory remained stable in statistical properties despite of drastic changes in case count and affect level. (A) Recognition memory performance, measured by mean d prime sensitivity index, did not change with respect to the time of data collection, even as COVID-19 cases in the US changed drastically. (B) Similar to our other measures of memory, the variance of the d prime index was also stable across the five data collection periods.

## Data Availability

Materials and data are available through Open Science Framework (https://osf.io/hywkt/).

## References

[CR1] Bangasser DA, Shors TJ (2007). The hippocampus is necessary for enhancements and impairments of learning following stress. Nature Neuroscience.

[CR2] Brady TF, Konkle T, Alvarez GA, Oliva A (2008). Visual long-term memory has a massive storage capacity for object details. Proceedings of the National Academy of Sciences of the United States of America.

[CR3] Brady TF, Konkle T, Gill J, Oliva A, Alvarez GA (2013). Visual long-term memory has the same limit on fidelity as visual working memory. Psychological Science.

[CR4] Brose A, Lövdén M, Schmiedek F (2014). Daily fluctuations in positive affect positively co-vary with working memory performance. Emotion.

[CR5] De Leeuw JR (2015). jsPsych: A JavaScript library for creating behavioral experiments in a Web browser. Behavior Research Methods.

[CR6] De Quervain D, Schwabe L, Roozendaal B (2016). Stress, glucocorticoids and memory: Implications for treating fear-related disorders. Nature Reviews Neuroscience.

[CR7] Dean DJ, Tso IF, Giersch A, Lee HS, Baxter T, Griffith T, Song L, Park S (2021). Cross-cultural comparisons of psychosocial distress in the USA, South Korea, France, and Hong Kong during the initial phase of COVID-19. Psychiatry Research.

[CR8] Faul F, Erdfelder E, Lang A-G, Buchner A (2007). G*Power 3: A flexible statistical power analysis for the social, behavioral, and biomedical sciences. Behavioral Research Methods.

[CR9] Fukuda K, Woodman GF (2015). Predicting and Improving Recognition Memory Using Multiple Electrophysiological Signals in Real Time. Psychological Science.

[CR10] Hidalgo V, Pulopulos MM, Puig-Perez S, Espin L, Gomez-Amor J, Salvador A (2015). Acute stress affects free recall and recognition of pictures differently depending on age and sex. Behavioural Brain Research.

[CR11] Hirst W, Phelps EA, Meksin R, Vaidya CJ, Johnson MK, Mitchell KJ, Buckner RL, Budson AE, Gabrieli JDE, Lustig C, Mather M, Ochsner KN, Schacter D, Simons JS, Lyle KB, Cuc AF, Olsson A (2015). A ten-year follow-up of a study of memory for the attack of September 11, 2001: Flashbulb memories and memories for flashbulb events. Journal of Experimental Psychology: General.

[CR12] Kensinger E, Baroff-Eaton RJ, Schacter D (2007). How negative emotion enhances the visual specificity of a memory. Journal of Cognitive Neuroscience.

[CR13] Levine LJ, Burgess SL (1997). Beyond general arousal: Effects of specific emotions on memory. Social Cognition.

[CR14] Milliken B, Jolicoeur P (1992). Size effects in visual recognition memory are determined by perceived size. Memory & Cognition.

[CR15] Needell, C. D., & Bainbridge, W. A. (2021). *Embracing new techniques in deep learning for estimating image memorability*. pp. 1–27. http://arxiv.org/abs/2105.10598.

[CR16] Ratcliff R, Smith PL, Brown SD, McKoon G (2016). Diffusion decision model: Current issues and history. Trends in Cognitive Sciences.

[CR17] Roozendaal B, McEwen BS, Chattarji S (2009). Stress, memory and the amygdala. Nature Reviews Neuroscience.

[CR25] Rouder JN, Speckman PL, Sun D, Morey RD, Iverson G (2009). Bayesian t-tests for accepting and rejecting the null hypothesis. Psychonomic Bulletin & Review.

[CR18] Shields GS, Doty D, Shields RH, Gower G, Slavich GM, Yonelinas AP (2017). Recent life stress exposure is associated with poorer long-term memory, working memory, and self-reported memory. Stress.

[CR19] Souza AS, Thaler T, Liesefeld HR, Santos FH, Peixoto DS, Albuquerque PB (2021). No evidence that self-rated negative emotion boosts visual working memory precision. Journal of Experimental Psychology: Human Perception and Performance.

[CR20] Watson D (1988). Intraindividual and interindividual analyses of positive and negative affect: Their relation to health complaints, perceived stress, and daily activities. Journal of Personality and Social Psychology.

[CR21] Watson D, Clark LA, Tellegen A (1988). Development and validation of brief measures of positive and negative affect: The PANAS scales. Journal of Personality and Social Psychology.

[CR22] Xie W, Zhang W (2016). Negative emotion boosts quality of visual working memory representation. Emotion.

[CR23] Xie W, Zhang W (2017). Negative emotion enhances mnemonic precision and subjective feelings of remembering in visual long-term memory. Cognition.

[CR24] Zhao C, Woodman GF (2021). Converging evidence that neural plasticity underlies transcranial direct-current stimulation (tDCS). Journal of Cognitive Neuroscience.

