# Supplementary material for: Even affective changes induced by the global health crisis are insufficient to perturb the hyper-stability of visual long-term memory
Source: Cogn Res Princ Implic. 2022 Jul 16;7:62. doi: 10.1186/s41235-022-00417-2 (PMC9287693; doi:10.1186/s41235-022-00417-2)
Supplement: Supplementary file 1 — Additional file 1. Fig. S1. Visual recognition memory remained stable in statistical properties despite of drastic changes in case count and affect level. (A) Recognition memory performance, measured by mean d prime sensitivity index, did not change with respect to the time of data collection, even as COVID-19 cases in the US changed drastically. (B) Similar to our other measures of memory, the variance of the d prime index was also stable across the five data collection periods. [file 41235_2022_417_MOESM1_ESM.docx]

**Additional file 1: Figure**
